# Temperature variability implies greater economic damages from climate change

**DOI:** 10.1038/s41467-020-18797-8

**Published:** 2020-10-06

**Authors:** Raphael Calel, Sandra C. Chapman, David A. Stainforth, Nicholas W. Watkins

**Affiliations:** 1 McCourt School of Public Policy, Georgetown University, Washington, DC USA; 2 Department of Physics, University of Warwick, Coventry, UK; 3 Grantham Research Institute on Climate Change and the Environment, London School of Economics and Political Science, London, UK; 4 Centre for the Analysis of Time Series, London School of Economics and Political Science, London, UK; 5 Faculty of Science, Technology, Engineering and Mathematics, Open University, Milton Keynes, UK

**Keywords:** Climate-change impacts, Climate-change impacts

## Abstract

A number of influential assessments of the economic cost of climate change rely on just a small number of coupled climate–economy models. A central feature of these assessments is their accounting of the economic cost of epistemic uncertainty—that part of our uncertainty stemming from our inability to precisely estimate key model parameters, such as the Equilibrium Climate Sensitivity. However, these models fail to account for the cost of aleatory uncertainty—the irreducible uncertainty that remains even when the true parameter values are known. We show how to account for this second source of uncertainty in a physically well-founded and tractable way, and we demonstrate that even modest variability implies trillions of dollars of previously unaccounted for economic damages.

## Abstract (web summary)

The authors estimate the damages associated with global temperature variability. They find that variability in temperature leads to substantial uncertainty about damages, which imposes costs equivalent to a large fraction of annual consumption today.

## Introduction

**Fig. 1:**
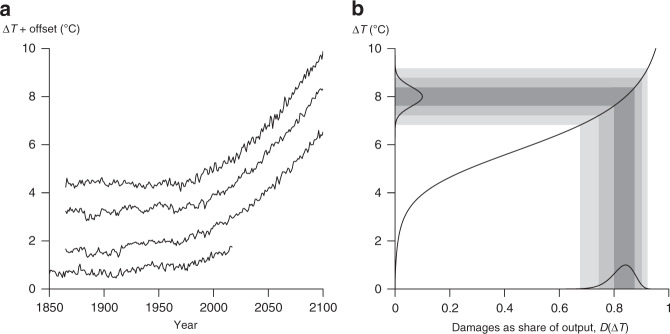
Temperature variability results in uncertain climate damages. **a** Observed and global circulation model simulated temperature trajectories are plotted here to illustrate the inter-annual variability typically present. From bottom up, the plot includes HadCRUT (observation), run 9 of EC-EARTH-4, run 1 of ACCESS1-0, and run 1 of CMCC-CMS (the last three being members of the CMIP5 ensemble). The four time series are offset to make the nature of the variability easier to see. **b** Reflects an illustrative normal distribution of the global mean temperature anomaly (*μ* = 8 and *σ* = 0.4) through Weitzman’s damage function^15^ to obtain economic damages as a share of global economic output. The shading traces the  ±*σ* range,  ±2*σ*, and  ±3*σ* of the temperature distribution, and shows that the damage distribution becomes left tailed when the expected value of global mean temperature is sufficiently high. It becomes right tailed when the expected value is lower.

Historical time series of global mean temperatures (GMT) exhibit substantial variability on annual, decadal, and longer timescales^1–3^ (Fig. 1). Global climate models also exhibit substantial internal variability, although less than in the historical record on longer timescales^1^. This variability means that even if we were able to accurately predict the expectation of GMT in each year (the ensemble mean over a collection of trajectories with different realisations of internal variability), our forecast trajectory would almost certainly miss the mark in any given year.

Although parts of the scientific^4^ and economic^5^ communities have long recognised that the climate may be better represented as a stochastic system, official assessments of climate damages still rely on models with deterministic GMT^6–8^. Their deterministic models generate trajectories of the expectation of GMT. These assessments can therefore account for uncertainty in the expected response to changing greenhouse gas concentrations by varying model parameters, but by design they omit that part of our uncertainty that arises from the deviations of individual realistic trajectories from the trajectory of the expectation.

To understand the economic consequences of this omission, start by noting that the annual GMT anomaly, Δ*T*, is the main input into the calculation of the economic damages from climate change. The annual economic damages from climate change, measured as a share of global annual output, are typically calculated by passing Δ*T* through a non-linear damage function (Fig. 1). The annual damages are then discounted and summed across all future years to give an estimate of total economic damages from climate change (Supplementary Note 5).

Notice that, if Δ*T* in any given year is represented by a distribution then this calculation produces a distribution of climate damages instead of a single-valued forecast (Fig. 1). Furthermore, the greater the positive autocorrelation of Δ*T*, the wider the distribution of the discounted and summed estimates of total economic damages (Supplementary Note 8).

**Fig. 2:**
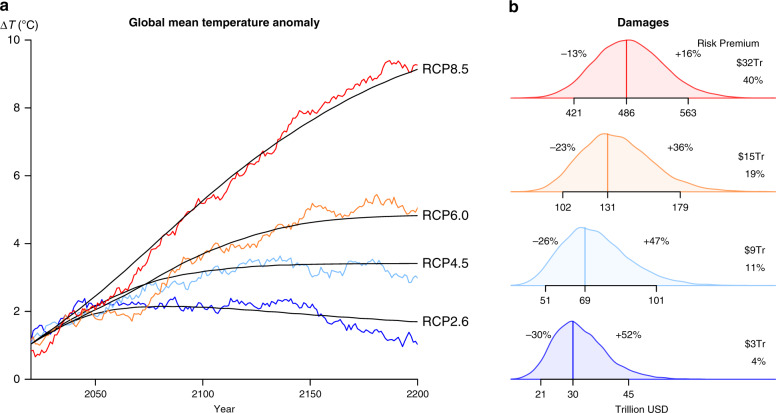
Damages with deterministic and stochastic temperatures. **a** Temperature trajectories produced by the deterministic model (in black) and from a single run of the stochastic model (in colour) with forcings taken from RCP8.5, 6.0, 4.5, and 2.6 (from top to bottom). We assume *C* = 10^9^ Jm^−2^K^−1^, *λ* = 1.23 Wm^−2^K^−1^, and *σ*_*Q*_ = 0.9375 × 10^8^ Wm^−2^s^½^, to approximate historical variability. **b** The distributions of damages obtained from ensembles of 10,000 temperature trajectories and a discount rate of 4.25% (to match^16^). The deterministic damages as well as the 5–95% range for the damage distribution are noted along the axis. The risk premia are included along the right edge, telling us what the canonical social planner (also with a 4.25% discount rate) would in principle be willing to pay to avoid inter-annual temperature variability, measured both in current dollars and as a share of current global output. See Supplementary Note 5 for a description of these calculations, Supplementary Note 6 for a discussion of the parameter choices, and Supplementary Note 7 for sensitivity analysis.

To capture this type of uncertainty, we have to extend the standard deterministic model. The simplest physical model that describes the stochastic behaviour of GMTs over time, and how this stochasticity depends on the underlying physical parameters, can be written as follows^4^:

1 $$C{\mathrm{d}}\Delta T=F{\mathrm{d}}t-\lambda \Delta T{\mathrm{d}}t+\sqrt{{\sigma }_{Q}^{2}{\mathrm{d}}t}\epsilon,$$

where *C* is the effective heat capacity, *F* the forcing, *λ* the feedback parameter, and ${\sigma }_{Q}^{2}$ is the variance of a zero-mean Gaussian noise process, *ϵ*. Figure 2 shows the different characteristics of the temperature time series produced when *C*, *λ*, and ${\sigma }_{Q}^{2}$ are calibrated to historical data (Supplementary Note 6), and also when ${\sigma }_{Q}^{2}$ is set to zero, as in the deterministic model. Note that the autocorrelation characteristics of this model can produce trajectories where many decades are above or below the deterministic trajectory (Fig. 2a).

We show, next, that adding realistic variability in global temperature in this way creates substantial uncertainty in future damages, equivalent to trillions of dollars of previously unaccounted for economic costs of climate change. The interaction of aleatory and epistemic uncertainty can further magnify these costs. Most of these costs cannot be avoided solely by strengthening mitigation policies at the margin. Our findings instead point to the conclusion that the benefits of adaptation are much greater than previously believed.

## Results

### Uncertainty about damages

Adding realistic temperature variability gives rise to substantial uncertainty in total climate damages (Fig. 2b). The deterministic model forecasts $486 trillion in total damages for the forcing scenario RCP8.5, but the stochastic model assigns a 5% chance to damages exceeding $563 trillion, 16% higher than the deterministic forecast. The 5–95% range for the stochastic model is (−13%, +16%) of the deterministic forecast. Lower forcings produce less warming, naturally, so the same amount of temperature variability produces greater relative dispersion of damages. For RCP2.6, for instance, the 5–95% range for the stochastic model is (−30%, +52%) of the deterministic forecast of $30 trillion.

### The risk premium

One conventional measure of the cost of this uncertainty is the so-called ‘risk premium’, also reported in Fig. 2. It is an answer to the question of what the canonical social planner would be willing to pay today to follow the deterministic temperature trajectory rather than to face an uncertain future. Another way to think about it is the value of insurance against aleatory uncertainty about future temperatures, if such an insurance product could be bought. The risk premium is calculated by evaluating the difference between the expected utility of the deterministic trajectory and that of the ensemble of stochastic trajectories (Supplementary Note 5). If the social planner knew to expect RCP2.6 forcings, for instance, she would today be willing to pay up to $3 trillion to eliminate just the internal variability, which is roughly 4% of current global output. A social planner that knew to expect RCP8.5 forcings would pay as much as $32 trillion, 40% of current output. Relative to the projections of the deterministic model, these risk premia represent anywhere from 6 to 13% in additional damages.

### Interacting risks

So far, we have only considered the cost of aleatory uncertainty while assuming fixed values of the model parameters. In practice, there is of course uncertainty about the deterministic trajectory as well, typically represented as uncertainty about the true values of the model parameters (epistemic uncertainty). One key parameter about which there is a great deal of uncertainty is the equilibrium climate sensitivity (ECS), which is inversely related to the feedback parameter *λ* at double CO_2_. Integrated assessment modellers typically capture this by rerunning the deterministic model for a sample of ECS values and producing a distribution of climate damages^7^. We can do the same thing with the stochastic model, which tells us how the cost of aleatory uncertainty changes when there is also epistemic uncertainty.

**Fig. 3:**
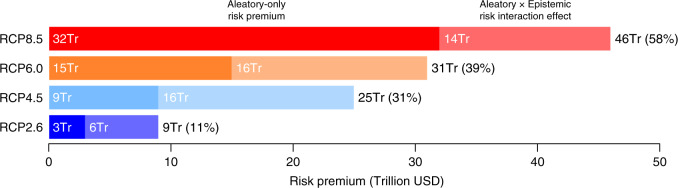
Aleatory uncertainty risk premia with and without underlying epistemic uncertainty. This plot shows what the canonical social planner would be willing to pay to avoid aleatory uncertainty when there is also underlying epistemic uncertainty. To compute these risk premia, we first obtain an ensemble of temperature trajectories by solving the deterministic EBM for a distribution of values of the equilibrium climate sensitivity (ECS). We assume that the ECS is log-normally distributed with a most likely value of 3 °C and Pr(2 ≤ ECS ≤ 4.5) = 0.66, in line with the IPCC’s fourth and fifth assessments, and otherwise uses the same physical assumptions as in Fig. 2. Next, we obtain a second ensemble by solving the stochastic energy balance model for the same distribution of ECS values. Both ensembles reflect the same epistemic uncertainty, but only the second incorporates aleatory uncertainty as well. The risk premia shown here are the difference between the expected utility of damages for the two ensembles (Supplementary Note 5). These risk premia can be decomposed into two parts: the darker portion of each bar shows the risk premium when all uncertainty is aleatory (same as in Fig. 2), while the lighter portion shows the additional risk premium arising from an interaction between aleatory and epistemic uncertainty. This risk interaction effect arises because a high draw from the ECS distribution produces both greater mean warming and greater variability, which makes the high draws disproportionately more costly. This results in damage distributions with a fatter right tail.

The risk premia reported in Fig. 3 show what the canonical social planner, faced with both epistemic and aleatory uncertainty, would be willing to pay to remove just the aleatory uncertainty. When faced with RCP2.6 forcings, a social planner that has epistemic uncertainty about the ECS, and aleatory uncertainty about the particular trajectory that would be realised for any given ECS, would be willing to pay $9 trillion to remove just the temperature variability, or roughly 11% of current global output. Faced with RCP8.5 forcings, she would be willing to pay as much as $46 trillion, over half of current output.

These risk premia are substantially higher than before as a result of how these two sources of uncertainty interact. In the stochastic model both the mean and the variance of the temperature are decreasing functions of *λ*, and thus increasing functions of the ECS deduced from *λ*. A high draw from the ECS distribution therefore produces both greater mean warming and greater variability (Supplementary Note 2). The effect of a higher ECS on the variance of temperature would be weaker if a fluctuation–dissipation theorem applied^9–11^, but it would not alter the fact that high draws become disproportionately more costly. This implies that the addition of stochasticity will produce damage distributions with an even fatter right tail than in the deterministic case. This is a distinct effect arising from the interaction of epistemic and aleatory uncertainties.

Epistemic uncertainty about the climate’s response, then, can magnify the cost of inter-annual and multi-decadal variability (Fig. 3). Since we cannot remove aleatory uncertainty, this risk premium is best thought of as a previously unaccounted for cost of climate change.

### The social cost of carbon

It is worth making special note of the distinction between the risk premia that we have estimated here and the marginal damage caused by releasing an additional tonne of CO_2_, the so-called social cost of carbon (SCC). The risk premium measures the additional cost of living with aleatory uncertainty as compared to living in a deterministic world. The SCC, by contrast, measures the cost of releasing just one more tonne of CO_2_ within a stochastic or deterministic framework. Even though the risk premium is substantial, we may well face the same temperature variability whether or not we release an additional tonne of CO_2_. Aleatory uncertainty is therefore unlikely to have much effect on the SCC (Supplementary Note 9). The crucial point to note is that the SCC and the risk premium provide answers to two different questions. The SCC tells us about costs that society can avoid through abatement of the marginal tonne of CO_2_. The risk premium primarily tells us about costs that we need to prepare for because they cannot be avoided in this way. The way to avoid them, rather, is through adaptation. The cost of adaptation is beyond the scope of the present investigation, but our findings suggest that the benefits are much greater than previously believed.

## Discussion

Adding temperature variability to a simple integrated assessment model results in greater economic damages from climate change. What is new here is neither the physics nor the economics—both of which closely follow canonical models in their respective fields—but we find that the careful combination of insights from these two disciplines reveals trillions of dollars of previously uncounted damages.

These damage estimates are substantial, but it is worth noting that they are likely to be on the conservative side. One reason for this is the typically high discount rate that is assumed for this type of analysis, which we have followed here (4.25%). If we relax this assumption, the damages from aleatory uncertainty become many times larger (Supplementary Note 7).

Another reason our estimates are conservative is the handling of temperature autocorrelation. The climate system represented by Eq. (1) is a continuous autoregressive process of the first order (Supplementary Note 2). It consequently exhibits autocorrelation, but it does not exhibit true long-range dependence, what has been termed the ‘Joseph effect’^12^ after the biblical story in which 7 years of plenty are followed by 7 years of famine. The autocorrelation in our model increases the probability of persistent events of this nature compared to a simple white noise time series, but if the climate system exhibits true long-range dependence^13^, long runs of extreme temperatures are even more likely than this simple model predicts. In this case, temperatures would be more likely to persistently deviate from the deterministic trajectory trend in one direction or the other, and the net present value of damages (plotted in Fig. 2) would be even more variable.

It should be noted, though, that this temperature persistence does not directly translate into higher risk premia for aleatory uncertainty in our analysis. The damage function and the social welfare function have no memory. It therefore only matters how the shape of the temperature distribution evolves over time, but it does not matter what the individual temperature trajectories look like that make up that evolving distribution. Autocorrelation gives rise to more extreme individual temperature trajectories, which widens the distribution of economic damages integrated over time, but this does not affect the risk premium (Supplementary Note 8). For the social planner envisioned by these integrated assessment models, then, the degree of autocorrelation of temperatures is largely irrelevant.

The simple integrated assessment models were built with a deterministic climate in mind, of course, so it is not entirely surprising that they are poorly equipped to deal with the consequences of temperature autocorrelation. But if societies lack the foresight to plan for longer periods of extreme climatic conditions, seven consecutive years of drought may be much more difficult to endure than if they were interspersed with years of plenty. In the story of Joseph, let us not forget, civilisation is saved only thanks to a divine prophecy. If, in reality, more strongly autocorrelated temperatures produce greater damages, then the true cost of temperature variability is likely to be substantially larger than we estimate here. More concretely, a country like Syria may be reasonably successful at containing the damage of a 1-year or even 2-year drought, but collapse under the weight of a 3-year drought, having far-reaching and disproportionate consequences. The combination of anthropogenic forcing and autocorrelated natural variability makes such severe droughts much more likely^14^. An important challenge in the years to come will be to pin down how damages accumulate during longer periods of extreme climate, so that these effects can be incorporated into future assessments of the economic damages from climate change.

## Supplementary information

Supplementary Information

## Data Availability

A full replication archive is available at 10.7910/DVN/5AJGH4.
