# Supplementary material for: Temperature variability implies greater economic damages from climate change
Source: Nat Commun. 2020 Oct 6;11:5028. doi: 10.1038/s41467-020-18797-8 (PMC7539010; doi:10.1038/s41467-020-18797-8)
Supplement: Supplementary file 1 — Supplementary Information [file 41467_2020_18797_MOESM1_ESM.pdf]

Supplementary Information for "Temperature variability implies greater economic damages from climate change"

Calel, Chapman, Stainforth, Watkins

Supplementary Note 1: The deterministic EBM

Several influential integrated assessment models forecast global mean temperatures using a simple energy balance model, or EBM [1]. The evolution of the global mean temperature anomaly, ΔT, is then described by the following deterministic ordinary differential equation [2, 3, 4].

$$C\frac{d\Delta T}{dt} = F(t) - \lambda\Delta T \tag{1}$$

C is the effective heat capacity per unit area (in $JK^{-1}m^{-2}$), $F(t)$ is the excess radiative forcing due to the perturbation (in Wm^{-2}), and λ is the feedback parameter (in $Wm^{-2}K^{-1}$).

Using Euler's method, the temperature at time $t + dt$ can be projected from values at t using the following update equation.

$$\Delta T(t + dt) = \left(1 - \frac{\lambda dt}{C}\right)\Delta T(t) + \frac{dt}{C}F(t) \tag{2}$$

Supplementary Note 2: The simplest stochastic EBM

In a seminal series of papers published in the mid-1970s, Hasselmann and colleagues introduced the concept of stochastic climate models [5, 6, 7], which has since grown into a mature discipline in its own right [8]. If their main purpose in incorporating stochasticity had been simply to express measurement uncertainty about the outcome of a deterministic EBM, it might have sufficed to add noise to their outputs. However, as [6] put it, they were in fact considering models where:

> ...slow changes of climate were interpreted as the integral response to continuous random excitation by short time scale "weather" disturbances.

This requires a model where fluctuations are incorporated directly into the evolution of the dynamical system.

This is now called the Hasselmann model, and is the simplest extension of equation 1 that captures the stochastic behaviour of global mean temperatures [5, 8]. It is an Itô stochastic differential equation (SDE):

$$Cd\Delta T = Fdt - \lambda\Delta T dt + \sqrt{\sigma_Q^2 dt}N_t^{t+dt}(0,1) \tag{3}$$

where σ_Q^2 represents the variance in the stochastic forcing associated with short-term variations in the heat fluxes at the Earth's surface; fluxes to and from the ocean, cryosphere, land surface systems and atmosphere. Here $\sqrt{dt}N_t^{t+dt}(0,1)$ is an explicit prescription [9] for the increment $dB(t)$ of the Wiener process (Brownian motion) over the time interval $(t, t + dt)$ [10], and we are following [5, 6] in assuming that these short term climate fluctuations can be described as white noise.

Beware that equation 3 cannot be divided through by the infinitesimal continuous time dt, as equation 1 can, since the temperature trajectory is no longer differentiable with respect to time. Rather, the change in ΔT between time t and $t + dt$ is now written as:

$$\Delta T(t + dt) - \Delta T(t) = \frac{\lambda}{C}\left(\frac{F(t)}{\lambda} - \Delta T\right)dt + \frac{\sigma_Q}{C}\sqrt{dt}N_t^{t+1}(0,1) \tag{4}$$

In the case of constant forcing, $F(t) = F$, the SDE has the solution [10]:

$$\Delta T(t) = e^{-\lambda t/C}\Delta T(0) + \frac{F}{C}\int_0^t e^{-\lambda(t-u)/C}du + \frac{\sigma_Q}{C}\int_0^t e^{-\lambda(t-u)/C}dB(u) \quad (5)$$

where $\int_0^t e^{-\lambda(t-u)/C}dB(u)$ is an Itô stochastic integral, illustrating that this solution is not simply the deterministic terms with arbitrary added noise. One can also see that the value of the Hasselmann time, C/λ, enters into the stochastic integral.

If the initial value $\Delta T(0)$ is a stochastic variable with mean F/λ and variance $\sigma_Q^2/2\lambda C$, the above solution is stationary [10]. However, since temperature trajectories are typically initialised at the current temperature, we will instead need the solution given a particular fixed initial value $\Delta T(0) = \Delta T_0$. This solution is nonstationary, and is a Normally distributed variable with time dependent conditional expectation value and conditional variance, $(\Delta T(t)|\Delta T(0) = \Delta T_0) \sim N(E(\Delta T|\Delta T(0) = \Delta T_0), Var(\Delta T|\Delta T(0) = \Delta T_0))$. When $\Delta T(0) = \Delta T_0$, the conditional expected value of ΔT at time t, the conditional variance at time t, and the autocorrelation function between times t and t' are given by [9, 10]:

$$\begin{aligned} E(\Delta T|\Delta T(0) = \Delta T_0) &= e^{-\lambda t/C}\Delta T_0 + \frac{F}{\lambda}(1 - e^{-\lambda t/C}) \\ Var(\Delta T(t)|\Delta T(0) = \Delta T_0) &= \frac{\sigma_Q^2}{2\lambda C}(1 - e^{-2\lambda t/C}) \\ ACF(\Delta T(t), \Delta T(t')|\Delta T(0) = \Delta T_0) &= \frac{e^{-\lambda|t-t'|/C} - e^{-\lambda(t+t')/C}}{\sqrt{(1 - e^{-2\lambda t/C})(1 - e^{-2\lambda t'/C})}} \end{aligned} \quad (6)$$

The first two expressions in equation 6 describe the ensemble mean and variance of the temperature anomaly distribution at fixed t, rather than time averaged quantities evaluated along a single temperature anomaly trajectory. The third is for an intrinsically two-point quantity, the autocorrelation function, again taken over an ensemble and evaluated between times t and t'.

This model provides a physically motivated way of understanding how temperature variability evolves over time. The temperature variance, for instance, depends on the heat fluctuation variance σ_Q^2, the effective heat capacity C, the feedback parameter λ, and time t, and the fact that σ_Q is constant does not constrain the temperature variance to be constant. As time goes on, the ensemble conditional mean, variance, and autocorrelation function all eventually "forget" the initial condition and tend to their stationary values of F/λ, $\sigma^2/(2\lambda C)$, and $\exp(-\lambda|t - t'|/C)$, respectively [10]. But while the global mean temperature is being forced to a new equilibrium level, the variance and autocorrelation are also changing.

Note, furthermore, that both the mean and variance of the temperature anomaly are decreasing functions of the feedback parameter, λ. A low value of λ, which corresponds to a high Equilibrium Climate Sensitivity (ECS) in this model, will therefore give rise to temperature trajectories with greater warming and variability. The effect of a higher ECS on the variance of temperature would be weaker if a fluctuation-dissipation theorem applied [11, 12, 13].

Supplementary Note 3: Numerically solving the EBMs

For constant F, we have seen that $\Delta T(t)$ is a Normally distributed random variable for the initial condition $\Delta T(0) = \Delta T_0$, i.e.

$$(\Delta T(t)|\Delta T(0) = \Delta T_0) = e^{-\lambda\Delta t/C}\Delta T_0 + \frac{F}{\lambda}(1 - e^{-\lambda\Delta t/C}) + \sqrt{\frac{\sigma_Q^2}{2\lambda C}(1 - e^{-2\lambda\Delta t/C})}N_0^t(0,1) \quad (7)$$

However, one cannot simulate the time series of conditioned $\Delta T(t)$ just by using this expression to generate a series of suitably scaled and displaced unit Normals, $N(0,1)$, at discrete time values $t, t + \Delta t, t + 2\Delta t, \ldots$ (see [9, pp. 57-58] and [14]). This procedure does incorporate the time dependence of the moments, but it does not preserve the desired two-time autocorrelation structure.

Instead we need an update equation, which is obtained by replacing ΔT_0 by $\Delta T(t)$ and using the conditional expression above to evolve the process over an interval of size Δt to obtain [9, 14]:

$$\Delta T(t + \Delta t) = e^{-\lambda\Delta t/C}\Delta T(t) + \frac{F}{\lambda}(1 - e^{-\lambda\Delta t/C}) + \sqrt{\frac{\sigma_Q^2}{2\lambda C}(1 - e^{-2\lambda\Delta t/C})}N_t^{t+\Delta t}(0,1) \quad (8)$$

This update equation has two of noteworthy properties. First, it is available at arbitrarily large time steps Δt (as noted explicitly by [14]). Second, time enters on the right hand side only once as a variable, effectively as a tag labelling $\Delta T(t)$, while all the other appearances of times are the chosen time step Δt. Since the time step is a constant, all the coefficients on the right hand side are also constants. In transitioning from the conditional value of a continuous stochastic process to the discrete update equation, then, we obtain an autoregressive process of the first order, relating $\Delta T(t+\Delta t)$ to the immediately previous $\Delta T(t)$. All that is required to get a standardised $AR(1)$ process is for Δt to be taken as 1 in the appropriate time units:

$$\Delta T_{t+1} = \phi \Delta T_t + \frac{F}{\lambda}(1-\phi) + \sqrt{\frac{\sigma_Q^2}{2\lambda C}(1-\phi^2)} N_t^{t+1}(0,1) \tag{9}$$

where we have written $\phi \equiv \exp(-\lambda \Delta t/C)$ at $\Delta t = 1$ to facilitate comparison with Supplementary Note 8. This is recognisable as an $AR(1)$ process with ϕ lying in the range 0 to 1 as required, with an additive constant of $(F/\lambda)(1-\phi)$, and a Gaussian white noise driver with variance $(\sigma_Q^2/2\lambda C)(1-\phi^2)$. From here on, we use "autocorrelation" to refer to the lag-1 autocorrelation coefficient, ϕ, rather than the general autocorrelation function.

The mapping between discrete $AR(1)$ and the continuous $AR(1)$ process that Gillespie's exact solver employs cannot be used when forcing is time dependent [14, 10]. Instead we use a simple and fast approximate approach, the Euler-Maruyama method [15], which combines evolution of the deterministic terms by an Euler scheme with a reasonable choice of a finer time step (in our case a month) to obtain acceptable accuracy in the stochastic integral.

The numerical algorithm we used was implemented with Matlab's `hwv` function (available through the Financial Toolbox), which solves the Hull-White and Vasicek models of interest rates. These are all cases of the SDE:

$$dX_t = S(t)[L(t) - X_t]dt + V(t)dB_t \tag{10}$$

where S, L and V represent the mean reversion Speed, mean reversion Level, and instantaneous Volatility rate, respectively, of the process variable X_t. Any or all of the parameters may be time dependent, and dB_t is an increment of Brownian motion. Comparison with equation 4 shows that in our case the solver is called with a constant $V = \sigma_Q/C$, a constant $S = \lambda/C$, and a time dependent $L(t) = F(t)/\lambda$.

Supplementary Note 4: EBMs and IAMs

Integrated assessment models (IAMs) couple simple climate models, like the one described in Supplementary Note 1, with simple economic models. These coupled models can be used to forecast the economic losses from climate change under a wide range of assumptions about physical and economic parameters, in order to assess the climate damages expected under particular climate policy proposals and to solve for optimal emissions trajectories that balance the expected costs and benefits of mitigation.

The social planner in these models is typically assumed to have a diminishing marginal utility from consumption. In a deterministic setting, this manifests as an aversion to inter-temporal inequality. If society is expected to be wealthier in the future than it is today, the social planner sees more value in a policy that transfers consumption from the wealthier future to the poorer present. A policy that foregoes current consumption to prevent future consumption losses is valued less by virtue of this inequality aversion.

The IAMs have also been extended to study different sources of consumption risk. In its investigations of climate risk specifically, the literature has been concerned primarily with epistemic uncertainty, represented as some probability distribution over the physical parameter values of an otherwise deterministic climate system. In the presence of this kind of epistemic uncertainty, the diminishing marginal utility from consumption not only implies an aversion to inter-temporal inequality, but an aversion to risk, too. Fundamentally, there are poorer and wealthier states of the world, and the canonical social planner makes no distinction whether these states are spread out in time or in probability space. As a consequence, the economic value of uncertain consumption is less than the value of the expectation (i.e. the mean) of consumption. Conversely, the economic cost

of uncertain climate damages is greater than the cost of the average of the damage distribution.[1] The Monte Carlo approach to representing climate risk has well-known limitations for calculating optimal policy trajectories [17], but it remains the dominant approach in official assessments of the economic damages from climate change [18].

The literature on recursive integrated assessment models have made significant advances in extending the economic analysis to consider a truly stochastic climate [19, 20]. [21] were the first to study a simple integrated assessment model in which there was uncertainty about both the values of the physical parameters and some inter-annual temperature variability. Their main concern was the ability of a Bayesian policy maker to make inferences about the parameter values of the climate system, with inter-annual variability serving primarily as a source of noise to muddy the signal. Their paper, and the literature it has spawned, has yielded many novel insights into how epistemic uncertainty affects the design of optimal greenhouse gas mitigation policy.

The approach to introducing inter-annual variability in this literature is to take a deterministic numerical model and add a Gaussian temperature shock with an exogenously given variance in each period. The Hasselmann model, which we employ here, has most in common with this approach but it differs in important respects. It represents a continuous stochastic process which imposes a relationship between the variance of temperature and other physical parameters, namely the heat capacity, C, the feedback parameter, λ, and the heat fluctuation variance σ_Q^2. When solved numerically it also imposes a relationship with the numerical timestep, Δt. This has many important benefits. For instance it takes account of the particular way that, conditional on the heat fluctuation variance σ_Q^2, a higher heat capacity (or lower climate sensitivity) would relate to smaller temperature fluctuations. By contrast, adding a Gaussian noise term with an exogenously given variance to the deterministic model does not preserve these physical relationships. Failing to take these relationships into account, a Bayesian policy maker might be at serious risk of drawing erroneous inferences about the climate parameters.

There are dangers in failing to appropriately capture the relationships between physical parameters in integrated assessment models [1], and in this respect, the Hasselmann model provides a significant and valuable refinement to current approaches. If it were incorporated into the economic literature on learning, it would allow the Bayesian policy maker to take account of the fact that the variance of temperature is jointly determined with the remaining parameters of interest. Still more significantly, it provides a minimal and physically appropriate stochastic representation of the climate that could be incorporated into the economic damage assessments that currently rely on simple deterministic climate models. In these damage assessments, even though the social planner is thought to dislike risk, and there uncertainty only about the parameter values of the physical model while the physical model used to forecast temperatures is fundamentally deterministic. Society faces no uncertainty about how temperatures will change from one year to the next.

Outside of these simple models, though, it is uncontroversial to observe that historical and forecast temperature time series of global mean temperatures show substantial inter-annual variability. The canonical social planner will dislike this additional source of uncertainty, and would be willing to pay to avoid it, for exactly the same reason that she dislikes uncertainty about the Equilibrium Climate Sensitivity (ECS), say. The expected damages resulting from a stochastic temperature trajectory will therefore be greater than those from a deterministic one.

Supplementary Note 5: Damages and the risk premium

In simple integrated assessment models, the damage function maps the global mean temperature onto the share of potential consumption that remains after taking into account the effects of climate change. In Nordhaus' canonical DICE model, the share of remaining consumption at time t is given by the following function of the concurrent global mean temperature anomaly, $\Delta T(t)$ [22].

$$D_N(\Delta T(t)) = \frac{1}{1 + 0.0023\Delta T(t)^2} \tag{11}$$

[1] Integrated assessment models have been extended to handle Epstein-Zin utility as well [16], an alternative social preference representation that does distinguish between inequality across time and across states of the world. Though these investigations often yield novel insights, our objective here is to assess the potential economic cost of inter-annual temperature variability in the standard framework. Our focus is therefore on how the canonical social planner would evaluate this source of consumption risk.

The parameter values of this damage function suggest an optimistic view of economic resilience, projecting that society will forfeit barely 19% of potential consumption in a 10°C warmer world.[2] Most accounts suggest that such significant warming would entail much more catastrophic losses, and attempts to calibrate damage functions with current economic data indicate that damages will be on the order of 20% to 75% of consumption at 5°C [23]. Weitzman proposes to address this by adding a higher order term to Nordhaus' quadratic function, which gives us the following damage function.[3]

$$D_W(\Delta T(t)) = \frac{1}{1 + \left(\frac{\Delta T(t)}{20.46}\right)^2 + \left(\frac{\Delta T(t)}{6.081}\right)^6} \tag{12}$$

Figure 1 plots Nordhaus' and Weitzman's damage functions side by side to visualise the differences. Note that even Weitzman's damage function predicts only a 27% loss of output for $\Delta T = 5$°C, which is at the lower end of the range suggested by empirical studies. Our main results in this paper are therefore computed using Weitzman's damage function.

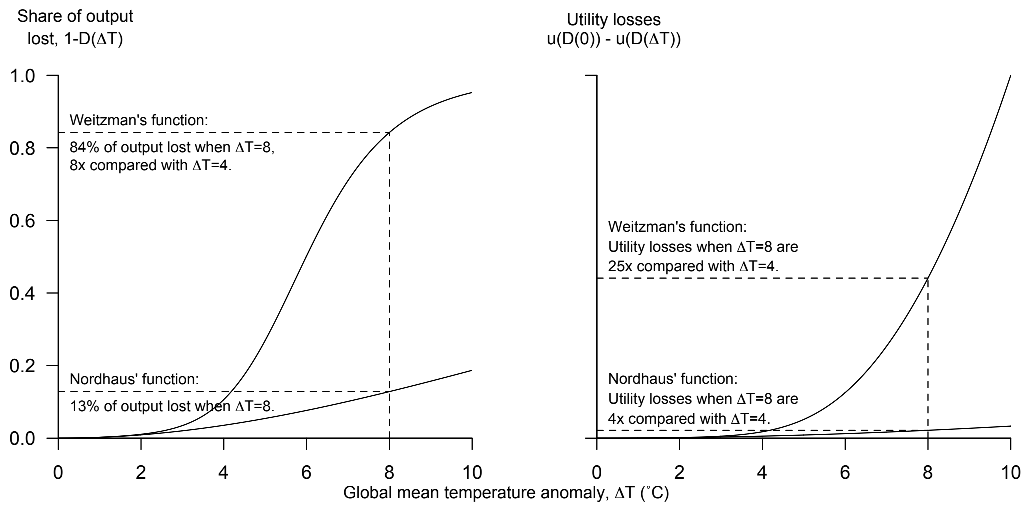

Supplementary Figure 1: **Translating temperatures into output losses and utility losses.** Panel (a) shows what share of output will be lost at different temperatures, according to Nordhaus' and Weitzman's damage functions. Panel (b) shows the utility losses associated with those damages, using the canonical social planner's utility function with $\eta = 1.45$.

To compute the monetary value of damages for time t, one multiplies $D(\Delta T(t))$ by the potential consumption in the absence of the effects of climate change, $C(t)$. To mirror DICE and several other models, we write $C(t)$ as the product of per capita consumption, $c(t)$, and the size of the population, $P(t)$, and evolve each separately. We assume that current per capita consumption, $c(0)$, would grow exponentially at a constant rate of g in the absence of climate change, and, as in DICE, that the population will grow according to the following difference equation.

$$P(t) = P(t-1)^{1-\alpha} P(\infty)^{\alpha} \tag{13}$$

$P(\infty)$ is the long-run asymptotic population level, and α governs the rate at which we approach the long-run value.

[2]It is worth clarifying that $\Delta T(t)$ is merely a summary statistic in this context, representing the state of the climate in a given year. It is a stand-in for the whole spatial pattern of temperatures, precipitation, and other weather variables. This means that even a constant ΔT is consistent with inter-annual variability at specific locations, indeed it presumes it as fact. When the damage function tells us what fraction of economic output would remain in a 10°C warmer world, then, it already accounts for the weather variability that is implied by that amount of global warming. This kind of local weather variability is therefore conceptually distinct from variability in the global mean temperatures itself, which the damage function takes no account of.

[3]In [24], the exponent on the higher order term is 6.754, chosen so that damages are 50% at 6°C and 99% at 12°. We round this exponent down to 6 here, since we require even-valued exponents to guarantee that damages are well-defined in the presence of inter-annual variability, which can send $\Delta T(t)$ below zero.

If the social planner discounts future consumption at a constant rate, r, we can then write the net present value of the stream of future consumption as follows.

$$\mathbb{C} = \sum_{t=0}^{T} \frac{1}{(1+r)^t}(1+g)^t c(0) D(\Delta T(t)) P(t) \tag{14}$$

The net present value of the stream of damages is just the difference between the stream of potential and actual consumption.

$$\mathbb{D} = \sum_{t=0}^{T} \frac{1}{(1+r)^t}(1+g)^t c(0)[1 - D(\Delta T(t))] P(t) \tag{15}$$

This expression gives us the monetary value of the economic damages from climate change associated with any temperature trajectory ΔT. We generate temperature trajectories using the Euler-Maruyama method outlined above, and the damage distributions plotted in figures 2 and 3 of the main paper are computed simply by feeding these ensembles of temperature trajectories through equation 15.

This simple damage calculation misses out an important feature of the IAMs, though, namely the social planner's diminishing marginal utility from consumption. The canonical planner is assumed to have preferences described by the following iso-elastic utility function.

$$u(t) = \frac{c(t)^{1-\eta}}{1-\eta} \tag{16}$$

where η is the elasticity of the marginal utility of consumption.[4] While consumption entered linearly before, it now enters through this concave utility function, which captures the social planner's increasing marginal disutility of damages. The first dollar in damages may not weigh heavily in our minds, but each successive dollar of damages matters more and more, and our bottom dollar is almost infinitely valuable. Figure 1 illustrates how temperatures translate into damages when the social planner exhibits diminishing marginal utility of consumption. When measured in terms of utility losses, damages rise at an increasing rate since the convexity of the utility function at low levels of consumption overpowers the concavity of the damage function at high temperatures.

When the planner is facing many possible global mean temperatures, indexed by s, each occurring with probability $p(t,s)$ at time t, the expected utility of consumption is given by the following weighted sum.

$$u(t) = \sum_{s} p(t,s) \frac{c(t,s)^{1-\eta}}{1-\eta} \tag{17}$$

As we have noted earlier, the elasticity of the marginal utility of consumption can in this setting be interpreted as a coefficient of inequality aversion as well as a coefficient of risk aversion.

The net present value of the stream of future utility from consumption for this social planner is written as follows.

$$\mathbb{C} = \sum_{s} \sum_{t=0}^{T} p(t,s) \frac{1}{(1+\rho)^t} \frac{[(1+g)^t c(0) D(\Delta T(t,s))]^{1-\eta}}{1-\eta} P(t) \tag{18}$$

ρ, sometimes known as the pure rate of time preference, substitutes for the discount rate r in this expression. We used r to represent the social planner's discount rate, but in this context the social planner will discount the future for two different reasons: (1) because she is impatient, here captured by ρ, and (2) because she prefers a little extra consumption in low-consumption years compared to high-consumption years, captured by η. The social discount rate is now a composite of these two considerations. Along an optimal consumption path, the social discount rate r is related to η and ρ by the following textbook formula: $r = \rho + g\eta$.

This expression gives us a different way to quantify the economic damages arising from interannual variability of the global mean temperature. When faced with a single deterministic temperature trajectory, $\overline{\Delta T}$, the net present value of the utility of consumption is $\mathbb{C}(\overline{\Delta T})$. Let us now

[4] As η goes to 1, this function approaches a logarithmic utility function.

imagine some distribution of temperature trajectories, $\widetilde{\Delta T}$, with a mean that is equal to the temperature trajectory $\overline{\Delta T}$ for every t. When faced with this distribution of temperature trajectories, the net present value of consumption is $\mathbb{C}(\widetilde{\Delta T})$. The disutility of inter-annual variability is then the difference between $\mathbb{C}(\widetilde{\Delta T})$ and $\mathbb{C}(\overline{\Delta T})$. If a risk-averse social planner could somehow secure a deterministic climate, her utility would increase by $\mathbb{C}(\widetilde{\Delta T}) - \mathbb{C}(\overline{\Delta T})$. Put another way, the social planner would be willing to pay a "premium" of as much as $\mathbb{C}(\widetilde{\Delta T}) - \mathbb{C}(\overline{\Delta T})$ to eliminate the risk that inter-annual variability entails. This quantity is therefore commonly referred to as the Risk Premium (RP).

This expression gives us the risk premium in units of utility, however, which are very unfamiliar and difficult to interpret. It is therefore helpful to normalise this difference by the value of a current marginal dollar, so that the risk premium can be understood in the same familiar units as the monetary damages calculated earlier.

$$RP = \frac{\mathbb{C}(\widetilde{\Delta T}) - \mathbb{C}(\overline{\Delta T})}{u(c(0)) - u(c(0) - 1)} \tag{19}$$

The risk premia reported in figure 2 of the main paper are calculated in this way. The same formula is used to compute the risk premia reported in figure 3 of the main paper, except that $\overline{\Delta T}$ is an ensemble of temperature trajectories generated by running the deterministic model for different values of the ECS, while $\widetilde{\Delta T}$ is an ensemble of trajectories generated by running the stochastic model for different values of the ECS.

In sum, we have reached two important and general conclusions so far. (1) Uncertainty about temperatures creates uncertainty about damages, (2) a social planner would be willing to pay a premium to avoid this uncertainty about future climate damages. Taken together, inter-annual variability implies greater economic damages from climate change than what has been assessed with deterministic EBMs.

Next, we select values for the physical and economic parameters of the model in order to gauge the economic significance of inter-annual temperature variability, which has not been directly quantified until now.

Supplementary Note 6: Choosing parameter values

Physical parameters

There are three key physical parameters in our model: the effective heat capacity C, the feedback parameter λ, and the variance of short-term disturbances in forcing σ_Q^2. As a starting point, a typical value quoted for C is 0.8×10^9 $Jm^{-2}K^{-1}$, with a $5-95\%$ uncertainty range of 0.2×10^9 to 2.0×10^9 [25]. With the forcing at two times CO_2 conventionally assumed to be 3.7 Wm^{-2} [26], $\lambda = 1.23$ corresponds to central ECS estimates of 3°C [27], which also corresponds well with the parameter values typically used in IAMs [1].

The parameter σ_Q is not typically estimated in the literature, so we must make our own selection. We choose values of C and λ roughly in line with central estimates, and then use the Euler-Maruyama method to simulate temperature trajectories for the period 1850 to 2015 for different values of σ_Q, initialised at $\Delta T_{1850} = 0$. A reasonable value of σ_Q will produce temperature trajectories with a similar time series properties as HadCRUT.

Figure 2 plots a few sample trajectories for the three different parameter combinations that come closest to reproducing the properties of the HadCRUT time series (shown on the bottom). When $C = 0.8 \times 10^9$ $Jm^{-2}K^{-1}$, a σ_Q of 0.625×10^8 $Wm^{-2}s^{1/2}$ will produce a similar time-averaged temperature variance as HadCRUT (panel a). However, there tends to be too little variability from year to year. Raising σ_Q in isolation results in an exaggerated temperature variance, so we must raise C in tandem. The additional heat capacity absorbs some of the additional variation. The combination of $C = 1.0 \times 10^9$ $Jm^{-2}K^{-1}$ and $\sigma_Q = 0.9375 \times 10^8$ $Wm^{-2}s^{1/2}$ appears to produce temperature trajectories that mimic HadCRUT both in terms of variance and autocorrelation (panel b). Increasing C and σ_Q further produces trajectories with realistic year-to-year variation, but more significant variance over a longer time horizon (panel c). Our main results are based on temperature trajectories simulated using $\{C = 1.0 \times 10^9, \lambda = 1.23, \sigma_Q = 0.9375 \times 10^8\}$, as shown in the panel (b), though we also conduct a sensitivity analysis below. Our forward-looking trajectories are initialised at $\Delta T_{2020} = 1$.

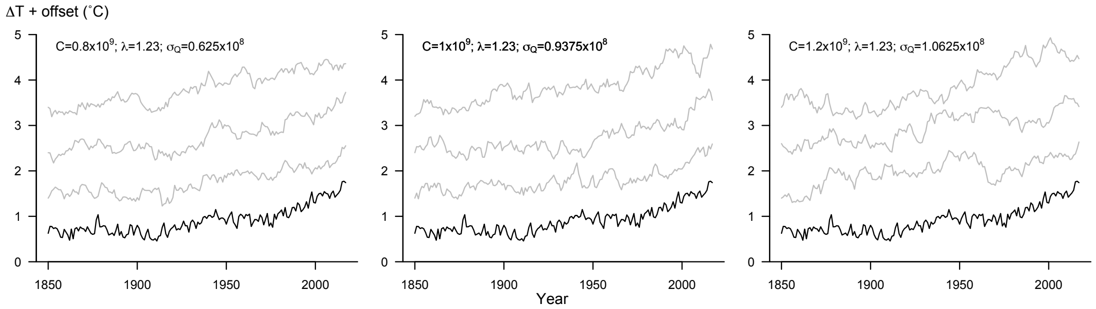

Supplementary Figure 2: **Choosing physical parameter values.** Different combinations of C, λ, and σ_Q produces trajectories that are more or less similar to historical temperatures. HadCRUT is shown in black at the bottom of panels (a), (b), and (c), while a sample trajectories from the stochastic EBM are plotted in grey. The time series are offset to make it easier to see the time series properties of each trajectory.

Finally, in figure 3 of the main paper we look at what happens to the risk premium when there is uncertainty about the ECS. Although there are many different estimates of the ECS, each associated with a different distribution [28], the IPCC reports a "likely" range (66% chance) from 2°C to 4.5°C and a most likely value of 3°C [27, 29]. Uncertainty about the ECS is perhaps best described by a fat-tailed distribution [30], but to be conservative we have fit a log-Normal distribution to the IPCC's modal value and likely range. We truncate the distribution at 20°C to ensure the expected values will converge. We then back out the corresponding distribution for the feedback parameter, λ, using a value for $F_{2\times CO_2} = 3.7Wm^{-2}$.

Economic parameters

To calculate economic damages and risk premia we must also choose values for a number of economic parameters. Since our objective is to quantify the damages as they would be assessed by IAMs, we have selected parameter values largely in line with these models. We adopt the assumption from Nordhaus' canonical DICE model [22] that the population grows from 7.5 billion to an asymptotic value of 11.5 billion at an annual rate of $\alpha = 0.0268$ (see previous section). We initialise the model at a per capita consumption level of $10,666 (based on a $80 trillion gross world product), and assume an underlying rate of consumption growth of $g = 1.9\%$. We conservatively assume that the social planner has a discount rate of $r = 4.25\%$, which many would consider a high discount rate in this context, but we do so to match the DICE model. In the case of a social planner with diminishing marginal utility, we follow DICE in assuming that $\rho = 1.5\%$ and $\eta = 1.45$. Our main departure from DICE is that we adopt Weitzman's [24] assumption of a higher-order term in the damage function, in order to bring us more in line with the recent empirical literature.

Supplementary Note 7: Sensitivity analysis

This paper is the first attempt at quantifying the economic damages from inter-annual temperature variability. We have tried to match our parametric assumptions to the scientific and economic literatures in an effort to better illustrate the magnitude of additional damages that would be added to conventional economic assessments of climate change if temperature variability was properly accounted for. Nevertheless, it is worth examining the sensitivity of our estimates to these assumptions.

Figure 3 replicates the damage distributions and risk premia for the three different $\{C, \lambda, \sigma_Q\}$-combinations shown in figure 2. A higher σ_Q increases the spread of the distribution, while a higher C shifts the distribution to the left. The combined effect is to increase the variance and skewness of the damage distribution, as well as the risk premium. In this particular case, the increase is small because the higher heat capacity nearly completely offsets the effect of the higher σ_Q. Reducing C and σ_Q, on the other hand, increases the expected damages, but reduces the risk premium.

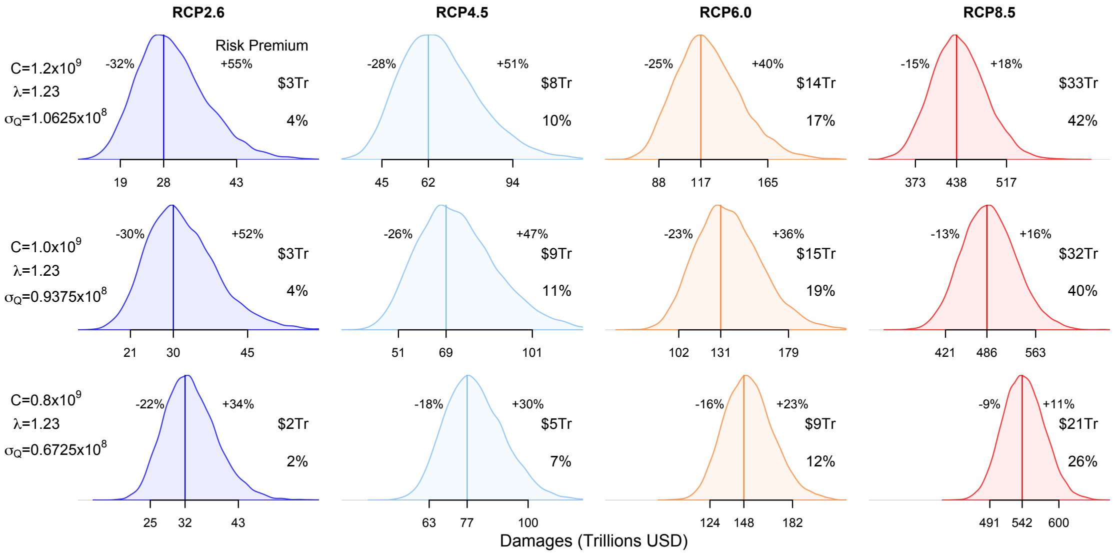

Supplementary Figure 3: **Sensitivity to physical parameter values.** The damage distributions and risk premia (reported in trillions of USD and as a share of current global output) are reported here for three different combinations of C, λ, and σ_Q that produce trajectories more or less similar to historical temperatures. The top row reports results for a higher C and σ_Q than the main paper, while the bottom row reports results for a lower C and σ_Q. A higher σ_Q increases a spread of the distribution, while a higher C shifts the distribution to the left.

Table 1 shows the risk premia under alternative representations of our uncertainty about the ECS. The top row makes the right tail thinner by truncating the log-Normal distribution at 10°C. This reduces the risk premia only very slightly. For comparison, the middle row reports the risk premia from the main paper. The bottom row shows the results for a Roe/Baker distribution [31, 32] which has a fatter right tail. Because uncertainty about the ECS is interacting with the inter-annual variability, greater tail risk increases the risk premium.

	RCP2.6	RCP4.5	RCP6.0	RCP8.5
IPCC log-Normal fit (<10°C)	9Tr (11%)	24Tr (30%)	31Tr (38%)	46Tr (58%)
IPCC log-Normal fit	9Tr (12%)	25Tr (31%)	31Tr (39%)	46Tr (58%)
Roe/Baker ($\bar{f} = 0.65, \sigma = 0.13$)	12Tr (15%)	28Tr (35%)	34Tr (43%)	51Tr (64%)

Supplementary Table 1: **Risk premia with different ECS distribution.** This table reports the risk premia associated with inter-annual variability in the presence of different background ECS uncertainty, both in terms of trillions of current dollars and as a share of current global output. For the Roe/Baker distribution, we compute the ECS distribution that obtains when their $\bar{f} = 0.65$, $\sigma = 0.13$, and $T_0 = 1.2$. These parameters do not directly correspond to the parameters in the stochastic EBM, but for the purpose of this sensitivity analysis we solve for the distribution of the feedback parameter λ that produces the same ECS distribution.

Tampering with the economic assumptions yields a much greater range of results. A social planner that is more patient (smaller ρ) will be willing to pay a substantially higher risk premium than in the canonical case (table 2). This is because she does not discount uncertainty about the far distant future as heavily. A more bearish social planner, expecting lower consumption growth, would likewise pay a higher risk premium. The reason is that a given amount of inter-annual variability leads to greater relative variability in consumption. A larger η, on its own, tends to reduce the risk premium, perhaps surprisingly. This is due to the dual role that η plays, increasing aversion to risk as well as to inter-temporal inequality. If the social planner is expecting robust

consumption growth, the aversion to inter-temporal inequality dominates and the social planner becomes less willing to pay now to save wealthier future generations from inter-annual variability. This effect dissipates in higher forcing scenarios in which future generations are not quite as well off.

Table 2 also shows how these features interact. A social planner that is both more patient and bearish would be much more willing to pay to avoid inter-annual variability. Greater risk aversion in a social planner that is either more patient or less bullish tends to reduce the risk premium in low forcing scenarios but magnify it in high forcing scenarios. A social planner that is more patient, less bullish, and more risk averse than the canonical case will be willing to pay a great deal more to avoid inter-annual variability. If faced with very high future forcings, she would be willing to pay many times the current level of global output to avoid just the variability.

$\{\rho,\eta,g\}$	RCP2.6	RCP4.5	RCP6.0	RCP8.5
Canonical case: {1.5%,1.45,1.9%}	3Tr (4%)	9Tr (11%)	15Tr (19%)	32Tr (40%)
Patient: {0.1%,1.45,1.9%}	9Tr (12%)	41Tr (52%)	98Tr (123%)	184Tr (230%)
Bearish: {1.5%,1.45,1%}	4Tr (5%)	12Tr (15%)	24Tr (30%)	49Tr (62%)
Risk averse: {1.5%,3,1.9%}	1Tr (1%)	2Tr (3%)	3Tr (4%)	37Tr (46%)
Patient and Bearish: {0.1%,1.45,1%}	15Tr (19%)	83Tr (104%)	212Tr (265%)	369Tr (461%)
Patient and Risk averse: {0.1%,3,1.9%}	2Tr (3%)	5Tr (6%)	9Tr (12%)	254Tr (317%)
Bearish and Risk averse: {1.5%,3,1%}	3Tr (3%)	7Tr (8%)	13Tr (17%)	468Tr (584%)
Patient, Bearish, and Risk averse: {0.1%,3,1%}	6Tr (8%)	22Tr (27%)	62Tr (78%)	5,857Tr (7,321%)

Supplementary Table 2: **Risk premia with different discount rates.** The discount rate is determined by the social planner's belief about the future growth rate of consumption in the absence of climate damages (g), her patience (ρ), and her marginal utility of consumption (η), the last of which can also be interpreted as risk aversion. This table reports the risk premia for different parameter combinations, both in trillions of current dollars and as a share of current global output. The canonical case from the main paper is reported in the first row.

Supplementary Note 8: The cost of temperature variance and autocorrelation

It is illuminating to parse out the economic consequences not just of the physical and economic parameter values individually, but also of the statistical moments of the temperature process. To do this, let us start by writing a simple AR(1) temperature process.

$$\Delta T(t+1) = a + \phi\Delta T(t) + \sigma_\epsilon \epsilon(t+1) \tag{20}$$

σ_ϵ is the standard deviation of a zero-mean white noise process ϵ, and $\|\phi\| < 1$. When the initial value, $\Delta T(0)$, is drawn from the same distribution as ϵ, this process is stationary and the mean, variance, and lag-1 autocorrelation of ΔT can be written as follows.

$$\begin{aligned} E(\Delta T(t)) &= \frac{a}{1-\phi} \\ Var(\Delta T(t)) &= \frac{\sigma_\epsilon^2}{1-\phi^2} \\ ACF(\Delta T(t), \Delta T(t-1)) &= \phi \end{aligned} \tag{21}$$

To understand how each affects the economic conclusions, we would like to be able to manipulate these moments independently. Notice that we can increase the mean by ratcheting up a without affecting the variance or autocorrelation. Similarly, we can increase the variance by turning up σ_ϵ^2 without affecting the mean or autocorrelation. However, if we increase the autocorrelation by raising ϕ, we simultaneously raise the mean and variance of the temperature process. To isolate the

pure effect of autocorrelation, we need to preserve the mean and variance of ΔT while ratcheting up ϕ. To do so, we need to re-scale the constant term as follows.

$$a^* = a(1-\phi) \tag{22}$$

We re-scale the standard deviation of the white noise as follows.

$$\sigma_\epsilon^* = \sigma_\epsilon\sqrt{1-\phi^2} \tag{23}$$

Each time we raise the autocorrelation, then, we also dampen drift and the white noise driver. This will produce temperature trajectories with the same mean and variance as each other but with different autocorrelations. Our re-scaled AR(1) temperature process can then be written as:

$$\Delta T(t) = a(1-\phi) + \phi\Delta T(t-1) + \sqrt{\sigma_\epsilon^2(1-\phi^2)}\epsilon(t) \tag{24}$$

This is exactly of the form of Gillespie's exact SDE solver, equation 9, and comparison with that reveals the following correspondences.

$$\begin{aligned} \phi &\equiv e^{-\lambda/C} \\ a &\equiv \frac{F}{\lambda} \\ \sigma_\epsilon^2 &\equiv \frac{\sigma_Q^2}{2\lambda C} \end{aligned} \tag{25}$$

This implies that there is a straightforward physical interpretation for each of our manipulations of the re-scaled AR(1) process. Raising the mean by raising a is equivalent to increasing forcing F. Raising the variance by raising σ_ϵ^2 is equivalent to increasing σ_Q^2. And raising the autocorrelation by raising ϕ is equivalent to manipulating the ratio of the feedback to the heat capacity, λ/C.

To look at the effect of temperature variance, first, it is actually sufficient to look at a single period. This permits us to isolate the effect of variance more cleanly, without confounding it with assumptions about the discount rate and the rate of population growth. We simply draw ΔTs from Normal distributions with different means and variances and feed them into the damage and utility functions. The result tells us how much the social planner would be willing to pay to avoid temperature uncertainty in a single year.

The logic of figure 1 (and indeed fig. 1 in the main paper) tells us that greater temperature variance in any single period will translate into greater variance of single-period damages. Since the social planner dislikes this variance, she would pay to avoid it if she could. Figure 4 shows how the risk premium for a single year changes as we move from the deterministic case (no variance) to greater and greater temperature variance. Three features are worth noting. First, as expected, the risk premium is increasing in the variance. Second, the risk premium is increasing at an increasing rate. Third, the rate of increase is greater at higher average temperatures. The marginal social cost of temperature variance, then, is greater when the average temperature is higher.

To look at the cost of autocorrelation, we use the re-scaled AR(1) process (equation 24) to draw a random sample of time series for $\Delta T(t)$, $\{\Delta T\}_t$, with different autocorrelations. We initialise the trajectories at $\Delta T_0 = a$.

First, let us consider how the temperature autocorrelation affects the distribution of damages (as plotted in figure 2 of the paper). Higher autocorrelations imply a greater risk of experiencing a run of hot or cold years. There is a greater likelihood of a near-term run of extreme temperatures, just as there is a greater likelihood of a more distant run of counter-balancing extreme temperatures. The variance of the temperature ensemble stays the same, then, but in the presence of discounting, the more distant temperature extreme does not completely offset the near-term extreme. A run of early hot years is therefore associated with a higher net present value of damages, even if a run of cold years eventually occurs. Conversely, a run of early cold years is associated with a lower net present value of damages, even if a run of hot years occurs later. As a result, autocorrelation increases the chances of a high or low draw of damages. Even though the temperature ensemble is identical at every point in time, the correlation across time increases the variance of the net present value of damages.[5]

[5]Like discounting, economic growth and population growth treat the near and distant future differently, and therefore break the symmetry that preserves the variance of the temperature ensemble. Discounting, economic growth, and population growth all induce a positive relationship between the autocorrelation of the temperature ensembles and the variance of the damage distribution.

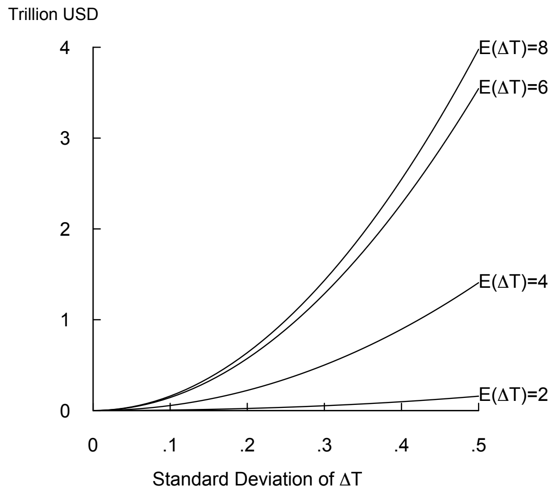

Supplementary Figure 4: **Single-year risk premium for different temperature means and variances.** This figure shows the risk premium calculated for a single year in which global consumption is $80 trillion and the world population is 7.5 billion, matching the initial values in our other simulations.

Figure 5 shows how the standard deviation of the net present value of damages varies as we ratchet up the autocorrelation of the temperature process. The net present value of damages is an increasing function of autocorrelation, as expected, except at the top end. The reason for this reversal is that it takes noticeably longer for the temperature ensemble to spread out from the fixed initial value when the autocorrelation is extremely high, so the near-term variance of the temperature ensemble itself actually declines significantly.[6] This effect vanishes if we initialise the temperature process at a random value instead of a fixed one.[7] A second feature of figure 5 is that the standard deviation of damages is initially increasing in the mean temperature, but then declines at temperature continues to rise. This is simply because there is less output left at such high temperatures (recall fig. 1).

The risk premium, also shown in figure 5, remains an increasing function of mean temperatures, as the convexity of the utility function overwhelms the concavity of the damage function at high temperatures. Perhaps surprisingly, though, autocorrelation does not appear to affect the risk premium. This is because the risk premium is calculated in two steps: first compute the utility from consumption in each period and state of the world, and then calculate the time-weighted and probability-weighted average. Autocorrelation will increase the variance of single-trajectory utilities, just as it increases the variance of single-trajectory damages, as we have just seen. But in neither case does it alter the mean of the distribution, and the expected utility is fundamentally the mean of single-trajectory utilities. From the perspective of the canonical social planner, then, autocorrelation does not matter. The risk premium is the same whether the autocorrelation is 0.9 or 0.1. The only reason why there is a gradient at all is that when the temperature process is initialised at a fixed value, a higher autocorrelation reduces the variance of the temperature ensemble in the initial years (see above), which the social planner prefers. This effect only grows large enough to make itself clearly known at very high autocorrelations.

It is easy to verify that the social planner is indifferent to autocorrelation itself by initialising the temperature process at a random value instead of a fixed one. A simple thought experiment illustrates the conceptual point. Imagine two ensembles. The first consists of two equally likely members, one with seven cold years followed by seven hot years, and the other with seven hot years

[6] The re-scaled AR(1) process (equation 24) is increasingly dominated by its deterministic component as ϕ approaches one. Put another way, it takes longer for the process to "forget" its initial value, so it takes longer for the individual trajectories to spread out from the initial value, a, as we move to cases with very high autocorrelation. As ϕ goes to one in the limit, the AR(1) does not collapse to the deterministic model, however, but instead becomes a nonstationary random walk. The deterministic case occurs when $\sigma_\epsilon^2 = 0$ irrespective of the value of ϕ.

[7] This is accomplished by running the AR(1) process for more periods and discarding the first part of the time series.

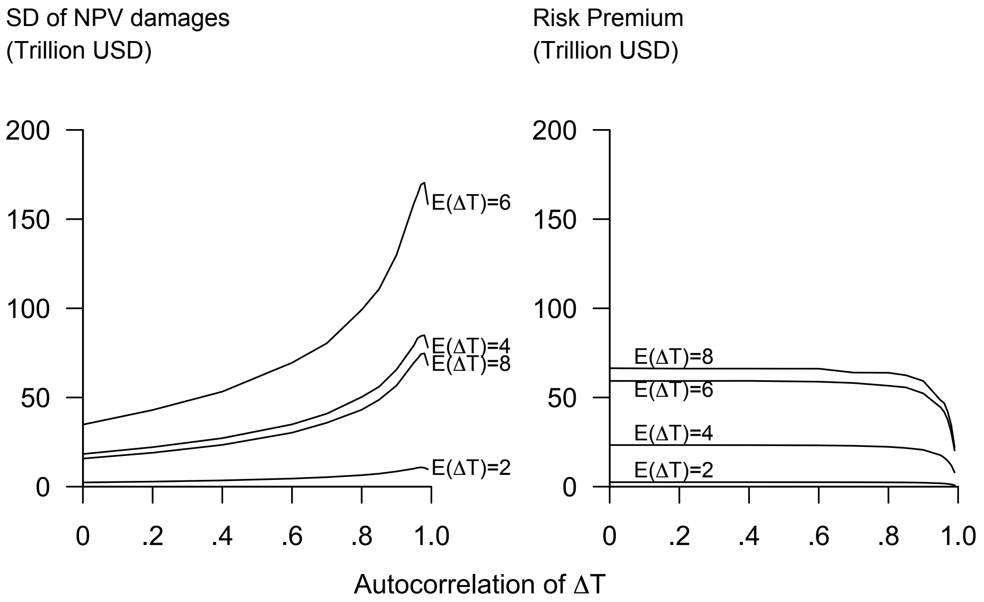

Supplementary Figure 5: **Standard deviation of damage distribution and risk premiums for different temperature autocorrelations.** The panel (a) shows how the standard deviation of the distribution of the net present value of damages changes as a function of autocorrelation, holding the mean of the temperature process constant at 2°C, 4°C, 6°C, and 8°C, and with an asymptotic standard deviation of 0.4. Since the temperature trajectories are initialised at the mean, the near-term variance of the temperature ensemble falls noticeably when the autocorrelation is large. The panel (b) shows how the risk premium varies with the temperature autocorrelation, conditional on the asymptotic mean and variance of the temperature process.

followed by seven cold years (fig. 6). The second ensemble consists of two equally likely members as well, each with fourteen randomly alternating hot and cold years (fig. 6). The spread of the net present value of damages is higher in the first ensemble than the second. But the risk premiums are calculated as a probability-weighted sum of the discounted single-period utilities, and in this example the set of temperatures, the probabilities, and the discount factors are identical across the two ensembles. The two ensembles merely relabel the terms in the sum as belonging to one member or the other. Economic damages, as defined in these models, do not depend on the autocorrelation of temperatures.

One can draw one of two conclusions from this. One is that it will be possible to account for the additional damages from temperature variability without making the integrated assessment model stochastic. If the social planner is truly indifferent to autocorrelation, it is at least technically possible to account for the extra damages by tweaking the parameters of the damage function, once the extra damages are known. Instead of each temperature mapping to the share of output that is lost at that specific temperature, it could be made to map to some larger share of output that is lost when that is the average temperature. This acknowledges that each level of the global temperature is in fact associated with some uncertainty, which translates in to a larger loss in the eyes of the social planner. The analysis we have presented here with a stochastic model is a necessary step to learn how large the re-calibration needs to be, but the structure of the deterministic IAM could ultimately be preserved.

Alternatively, one might conclude that our analysis has revealed a significant shortcoming in how IAMs estimate climate damages. Damages in the real world tend to accumulate non-additively, such that a run of extreme temperatures produce disproportionate social and economic consequences. To incorporate this into the IAMs, it is not enough merely to tweak the parameters of the damage function. Rather one must make damages an explicit function of present and past temperatures. An integrated assessment model with path dependent damages would not treat autocorrelation as benign or irrelevant, but would recognise that it can cause significant harm. Such a model would likely project much greater economic damages from climate change than we have found here. There are many conceptual and empirical challenges that will need to be

overcome to accomplish such a significant reformulation of the damage function, though, enough to constitute an ambitious research agenda for years to come.

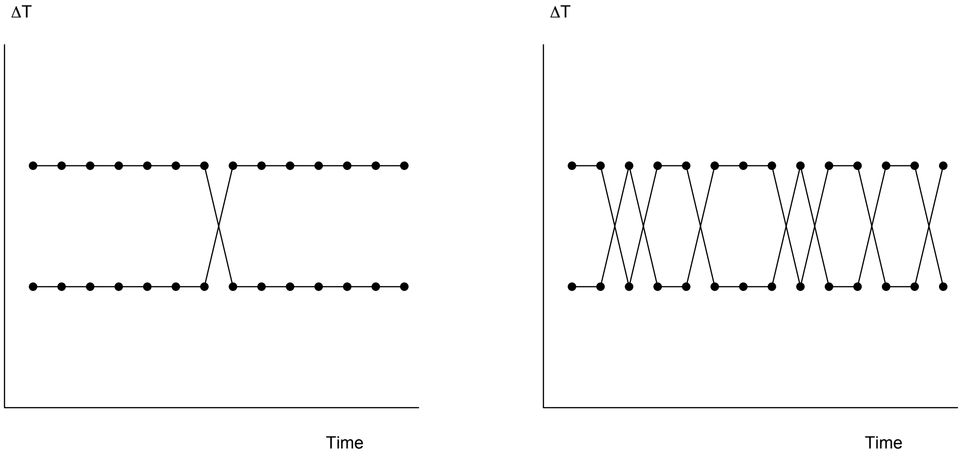

Supplementary Figure 6: **Two temperature ensembles that differ only with respect to autocorrelation.** The panel (a) shows a temperature ensemble with high autocorrelation, while the panel (b) shows a temperature ensemble with low autocorrelation. The mean and variance is identical in both ensembles.

Supplementary Note 9: A note on the social cost of carbon

Some readers may be curious about what our results imply for the social cost of carbon (SCC)—a quantity that is often estimated with integrated assessment models. It is worth observing, first, that there is an important distinction between the risk premium that we estimate and the SCC. The risk premium measures the additional costs of living with aleatory uncertainty as compared to living in a deterministic world, while the SCC measures the additional cost of releasing one more tonne of CO_2 *within* a stochastic or determistic framework. Even though the cost of aleatory uncertainty may be substantial, society incurs that cost whether or not one more tonne of CO_2 is released. Aleatory uncertainty is therefore unlikely to have much effect on the SCC.

Still, aleatory uncertainty will likely result in a slightly greater SCC. An extra tonne of CO_2 increases radiative forcing, F, which raises mean temperatures, and, as shown in fig. 4, this increases the damages of a given amount of temperature variability. This is precisely why the risk premium is larger for RCP4.5 than for RCP2.6, say. Further, one could also imagine that an extra tonne of CO_2 might increase σ_Q, which would further increase the damanges and the SCC.

So far we have taken F as an input and assumed that σ_Q is fixed. All of our conclusions thus far therefore hold under any carbon cycle. To estimate the extra economic damages from releasing an additional tonne of CO_2, however, one must venture a specific model of how emissions affect these quantities.

It is no easy task providing a credible model of the link between a marginal tonne of CO_2 and temperature, inclusive of the epistemic and aleatory uncertainties present in the carbon cycle itself. We do not attempt it here. Rather, we offer a simplified calculation to give readers a general sense of the magnitude of the effect, but we also request that the result be interpreted with appropriate caution.

For the purpose of illustration, then, let us assume that emissions have no effect on σ_Q, and that the fraction of a pulse of CO_2 emissions that remains in the atmosphere in year t is given by the following one-equation carbon cycle model [33].

$$AF_t = 0.217 + 0.259e^{-t/172.9} + 0.338e^{-t/18.51} + 0.186e^{-t/1.186}$$

We then imagine a 100 $GtCO_2$ pulse in 2020 and calculate how much of it will remain in the atmosphere over time. The associated increase in radiative forcing can be approximated by assuming an initial atmospheric stock of 3,198 $GtCO_2$ in 2020 (an atmospheric concentration of 410 ppm multiplied by a factor of 7.8[8]), and applying the logarithmic relationship between CO_2 and radiative forcing ($5.35 \ln([CO2_0 + 100GtCO2 \times AF_t]/CO2_0)$). Assuming that the underlying atmospheric concentration of CO_2 remains fixed at the initial level, the pulse gives us an increase in radiative forcing of about 0.16 W/m^2 in the first year, and then declines gradually.

The SCC is typically calculated for a small departure from the optimal emissions trajectory, but we would need to add quite a bit more structure to solve for the optimal trajectory here. For simplicity, let us instead use RCP4.5 as an approximation of the optimal trajectory, noting that the temperature trajectory for RCP4.5 looks quite similar to recently published optimal temperature trajectories for DICE (e.g. [35]).

We can then add the radiative forcing associated with a 100 $GtCO_2$ pulse to the RCP4.5 forcing trajectory (call this new trajectory "RCP4.5+"). Since the atmospheric concentration of CO_2 rises under RCP4.5, the contribution to radiative forcing from a 100 $GtCO_2$ pulse will decline faster than under the assumption of a fixed concentration. We use RCP4.5+ here as a helpful approximation, but are also mindful that the difference between RCP4.5 and RCP4.5+ will somewhat overstate the effect of releasing additional CO_2.

We then recompute economic damages under RCP4.5+. Dividing the difference in economic damages between RCP4.5 and RCP4.5+ by 100 billion returns an estimate of the marginal damage from just one additional tonne of CO_2—the SCC.

In order for the results to be comparable to other studies, we have run an ensemble with both epistemic uncertainty and aleatory uncertainty (same as figure 3 in manuscript) for RCP4.5 and RCP4.5+, and computed the indicative SCC as a probability-weighted difference across the distribution of ECS. This gives us an estimate of the SCC in the deterministic model with epistemic uncertainty (this corresponds to how the SCC is computed in the official assessments we cite in the paper), as well as an estimate of the SCC in the stochastic model with epistemic uncertainty (i.e. with *both* types of uncertainty present). The difference between these two SCCs is a measure of the effect of aleatory uncertainty on the SCC.

We estimate that aleatory uncertainty increases the SCC by 0.75%. So, by specifying a simplified carbon cycle model, we see that our findings are in fact broadly consistent with existing literature.

The more important difference between our results and previous studies is that the risk premium and the SCC provide answers to two different questions. The SCC tells us about costs that society can avoid through abatement of a marginal tonne of CO_2. The risk premium primarily tells us about costs that we need to prepare for because they cannot be avoided in this way. The way to avoid them, rather, is through adaptation.

Supplementary References

[1] R. Calel and D. A. Stainforth, "On the Physics of Three Integrated Assessment Models," *Bulletin of the American Meteorological Society*, vol. 98, pp. 1199–1216, June 2017.

[2] D. G. Andrews and M. R. Allen, "Diagnosis of climate models in terms of transient climate response and feedback response time," *Atmospheric Science Letters*, vol. 9, no. 1, pp. 7–12, 2008.

[3] C. A. Senior and J. F. B. Mitchell, "The time-dependence of climate sensitivity," *Geophysical Research Letters*, vol. 27, no. 17, pp. 2685–2688, 2000.

[4] R. E. Dickinson, "How will climate change?," in *The greenhouse effect, climatic change and ecosystems. SCOPE Report* (B. Bolin, B. R. Döös, J. Jäger, and R. A. Warrick, eds.), vol. 29, pp. 206–270, John Wiley and Sons, Chichester, 1986.

[5] K. Hasselmann, "Stochastic climate models Part I. theory," *Tellus*, vol. 28, no. 6, pp. 473–485, 1976.

[6] C. Frankignoul and K. Hasselmann, "Stochastic climate models Part II. application to sea-surface temperature anomalies and thermocline variability," *Tellus*, vol. 29, no. 4, pp. 289–305, 1977.

[7] P. Lemke, "Stochastic climate models Part III. application to zonally averaged energy models," *Tellus*, vol. 29, no. 5, pp. 385–392, 1977.

[8] 1 ppm by volume of atmosphere CO_2 translates to 2.13 GtC, 1 part C translates to 3.664 parts CO_2, and $2.13 \times 3.664 = 7.8$. Conversion factors taken from [34].

[8] C. Franzke and T. O'Kane, eds., *Nonlinear and Stochastic Climate Dynamics*. Cambridge, UK: Cambridge University Press, 2017.

[9] D. S. Lemons, *An Introduction to Stochastic Processes in Physics*. Baltimore, Maryland: Johns Hopkins University Press, 2002.

[10] P. J. Brockwell and R. A. Davis, *An Introduction to Time Series and Forecasting, 3rd Edition*. Switzerland: Springer, 2016.

[11] P. M. Cox, C. Huntingford, and M. S. Williamson, "Emergent constraint on equilibrium climate sensitivity from global temperature variability," *Nature*, vol. 553, no. 7688, pp. 319–322, 2018.

[12] M. Rypdal, H.-B. Fredriksen, E. Myrvoll-Nilsen, K. Rypdal, and S. H. Sørbye, "Emergent scale invariance and climate sensitivity," *Climate*, vol. 6, no. 4, p. 93, 2018.

[13] D. P. Cummins, D. B. Stephenson, and P. A. Stott, "Optimal Estimation of Stochastic Energy Balance Model Parameters," *Journal of Climate*, 04 2020.

[14] D. T. Gillespie, "The mathematics of Brownian motion and Johnson noise," *American Journal of Physics*, vol. 64, no. 3, pp. 225–240, 1996.

[15] D. J. Higham, "An algorithmic introduction to numerical simulation of stochastic differential equations," *SIAM Review*, vol. 43, no. 3, pp. 319–322, 2001.

[16] K. D. Daniel, R. B. Litterman, and G. Wagner, "Declining co2 price paths," *Proceedings of the National Academy of Sciences*, vol. 116, no. 42, pp. 20886–20891, 2019.

[17] B. Crost and C. P. Traeger, "Optimal climate policy: Uncertainty versus monte carlo," *Economics Letters*, vol. 120, no. 3, pp. 552 – 558, 2013.

[18] Interagency Working Group, "Technical support document: Technical update of the social cost of carbon for regulatory impact analysis under executive order 12866," tech. rep., Interagency Working Group on Social Cost of Carbon, United States Government, 2015.

[19] C. P. Traeger, "A 4-stated dice: Quantitatively addressing uncertainty effects in climate change," *Environmental and Resource Economics*, vol. 59, pp. 1–37, Sep 2014.

[20] D. Lemoine and I. Rudik, "Managing climate change under uncertainty: Recursive integrated assessment at an inflection point," *Annual Review of Resource Economics*, vol. 9, no. 1, pp. 117–142, 2017.

[21] D. L. Kelly and C. D. Kolstad, "Bayesian learning, growth, and pollution," *Journal of Economic Dynamics and Control*, vol. 23, no. 4, pp. 491 – 518, 1999.

[22] W. D. Nordhaus, "Revisiting the social cost of carbon," *Proceedings of the National Academy of Sciences*, vol. 114, no. 7, pp. 1518–1523, 2017.

[23] M. Burke, S. M. Hsiang, and E. Miguel, "Global non-linear effect of temperature on economic production," *Nature*, vol. 527, pp. 235–239, Nov. 2015.

[24] M. L. Weitzman, "GHG Targets as Insurance Against Catastrophic Climate Damages," *Journal of Public Economic Theory*, vol. 14, no. 2, pp. 221–244, 2012.

[25] D. J. Frame, B. B. B. Booth, J. A. Kettleborough, D. A. Stainforth, J. M. Gregory, M. Collins, and M. R. Allen, "Constraining climate forecasts: The role of prior assumptions," *Geophysical Research Letters*, vol. 32, no. 9, p. L09702, 2005.

[26] G. Myhre, E. J. Highwood, K. P. Shine, and F. Stordal, "New estimates of radiative forcing due to well mixed greenhouse gases," *Geophysical research letters*, vol. 25, no. 14, pp. 2715–2718, 1998.

[27] S. Solomon, D. Qin, M. Manning, R. Alley, T. Berntsen, N. Bindoff, Z. Chen, A. Chidthaisong, J. Gregory, G. Hegerl, M. Heimann, B. Hewitson, B. Hoskins, F. Joos, J. Jouzel, V. Kattsov, U. Lohmann, T. Matsuno, M. Molina, N. Nicholls, J. Overpeck, G. Raga, V. Ramaswamy, J. Ren, M. Rusticucci, R. Somerville, T. Stocker, P. Whetton, R. Wood, and D. Wratt, "Technical summary," in *Climate Change 2007: The Physical Science Basis. Contribution of Working Group I to the Fourth Assessment Report of the Intergovernmental Panel on Climate Change* (S. Solomon, D. Qin, M. Manning, Z. Chen, M. Marquis, K. Averyt, M. Tignor, and H. Miller, eds.), Cambridge University Press, Cambridge, United Kingdom and New York, NY, USA, 2007.

[28] M. Meinshausen, N. Meinshausen, W. Hare, S. Raper, K. Frieler, D. Frame, and M. Allen, "Greenhouse-gas emission targets for limiting global warming to 2c," *Nature*, vol. 458, pp. 1158–1162, April 2009.

[29] T. Stocker, D. Qin, G.-K. Plattner, L. Alexander, S. Allen, N. Bindoff, F.-M. Bréon, J. Church, U. Cubasch, S. Emori, P. Forster, P. Friedlingstein, N. Gillett, J. Gregory, D. Hartmann, E. Jansen, B. Kirtman, R. Knutti, K. Krishna Kumar, P. Lemke, J. Marotzke, V. Masson-Delmotte, G. Meehl, I. Mokhov, S. Piao, V. Ramaswamy, D. Randall, M. Rhein, M. Rojas, C. Sabine, D. Shindell, L. Talley,

D. Vaughan, and S.-P. Xie, "Technical summary," in *Climate Change 2013: The Physical Science Basis. Contribution of Working Group I to the Fifth Assessment Report of the Intergovernmental Panel on Climate Change* (T. Stocker, D. Qin, G.-K. Plattner, M. Tignor, S. Allen, J. Boschung, A. Nauels, Y. Xia, V. Bex, and P. Midgley, eds.), pp. 33–115, Cambridge, United Kingdom and New York, NY, USA: Cambridge University Press, 2013.

[30] M. Weitzman, "On modeling and interpreting the economics of catastrophic climate change," *The Review of Economics and Statistics*, vol. 91, no. 1, pp. 1–19, 2009.

[31] G. H. Roe and M. B. Baker, "Why Is Climate Sensitivity So Unpredictable?," *Science*, vol. 318, pp. 629 –632, Oct. 2007.

[32] M. Baker and G. Roe, "The shape of things to come: why is climate change so predictable?," *Journal of Climate*, vol. 22, pp. 4574–4589, Jan 2009.

[33] P. Forster, V. Ramaswamy, P. Artaxo, T. Berntsen, R. Betts, D. W. Fahey, J. Haywood, J. Lean, D. C. Lowe, G. Myhre, *et al.*, "Changes in atmospheric constituents and in radiative forcing. chapter 2," in *Climate Change 2007. The Physical Science Basis*, 2007.

[34] F. M. O'Hara Jr., "Glossary: Carbon Dioxide and Climate," Tech. Rep. ORNL/CDIAC-39, Carbon Dioxide Information Analysis Center, Oak Ridge National Laboratory, Oak Ridge, Tennessee, Third Edition 1990.

[35] W. Nordhaus, "Projections and uncertainties about climate change in an era of minimal climate policies," *American Economic Journal: Economic Policy*, vol. 10, pp. 333–60, August 2018.
